# Effectiveness of classroom based crew resource management training in the intensive care unit: study design of a controlled trial

**DOI:** 10.1186/1472-6963-11-304

**Published:** 2011-11-10

**Authors:** Peter F Kemper, Martine de Bruijne, Cathy van Dyck, Cordula Wagner

**Affiliations:** 1 Department of Public and Occupational Health; EMGO+ Institute for Health and Care Research, VU Medical Center, Van der Boechorststraat 7, 1081 BT Amsterdam, The Netherlands; 2 Faculty of Social Sciences, Department of Organization Sciences, VU University, De Boelelaan 1081, 1081 HV Amsterdam, The Netherlands; 3 The Netherlands Institute of Health Services Research (NIVEL), Otterstraat 118, 3513 CR Utrecht, The Netherlands

## Abstract

**Background:**

Crew resource management (CRM) has the potential to enhance patient safety in intensive care units (ICU) by improving the use of non-technical skills. However, CRM evaluation studies in health care are inconclusive with regard to the effect of this training on behaviour and organizational outcomes, due to weak study designs and the scarce use of direct observations. Therefore, the aim of this study is to determine the effectiveness and cost-effectiveness of CRM training on attitude, behaviour and organization after one year, using a multi-method approach and matched control units. The purpose of the present article is to describe the study protocol and the underlying choices of this evaluation study of CRM in the ICU in detail.

**Methods/Design:**

Six ICUs participated in a paired controlled trial, with one pre-test and two post test measurements (respectively three months and one year after the training). Three ICUs were trained and compared to matched control ICUs. The 2-day classroom-based training was delivered to multidisciplinary groups. Typical CRM topics on the individual, team and organizational level were discussed, such as situational awareness, leadership and communication. All levels of Kirkpatrick's evaluation framework (reaction, learning, behaviour and organisation) were assessed using questionnaires, direct observations, interviews and routine ICU administration data.

**Discussion:**

It is expected that the CRM training acts as a generic intervention that stimulates specific interventions. Besides effectiveness and cost-effectiveness, the assessment of the barriers and facilitators will provide insight in the implementation process of CRM.

**Trial registration:**

Netherlands Trial Register (NTR): NTR1976

## Background

The risks, potential harm and costs of adverse events for patients at intensive care units (ICU) are larger than in other hospital departments [1], making improvement of patient safety in ICUs all the more important. The ICU is particularly vulnerable in terms of patient safety threats related to ineffective teamwork or failure to follow protocol [2,3]. Results of a Dutch record review study revealed that 9.4% of all patients admitted to the ICU experienced one or more adverse events, which is far above the average of a hospital (5.7%)[4]. Of these adverse events 50% were considered highly preventable. Similarly, Vincent and colleagues [5] reported 1.7 adverse events per patient per day in a medical-surgical ICU.

It has been demonstrated that unsafe care more often originates from problems with non-technical skills than from a lack of technical expertise [6,7]. Non-technical skills are 'the cognitive, social and personal resource skills that complement technical skills and contribute to safe and efficient task performance' [8]. For example to be explicit in coordinating tasks or to share information. Studies have shown that a lack of non-technical skills led to poor teamwork resulting in critical incidents in the ICU [9,10].

Both national and international health authorities have advocated crew resource management (CRM) as a method to improve non-technical skills, especially in emergency departments, surgery and intensive care [11-13]. CRM, which has its roots in high-risk industries such as aviation [14], was developed in the early 1980's as a response to the finding that unsafe flight conditions were frequently the result of failures in pilots' non-technical skills rather than a lack in technical knowledge [15]. It has been shown that CRM can effectively improve safety in a variety of professional domains, such as nuclear power and offshore oil production [16,17]. It is plausible that the general principles of CRM can be used in the ICU as well, because the ICU shares characteristics with workplaces where CRM has been proven to be effective, such as high-stake outcomes, complex actions, and high time pressure [18].

CRM is based on the premise that human error is avoidable, but can never be eradicated. It is typically directed at creating awareness of human factors and performance limiters [19], and at teaching behaviours to neutralize these threats, for instance through leadership and speaking up [15]. Furthermore, it forces participants to assess and think about personal and peer behaviour. Concepts that are introduced during the training include inquiry, seeking relevant task related information, advocacy, communicating proposed actions, conflict resolution and decision making [19-21]. Thus, CRM is directed at increasing awareness of human limitations and changing team behaviour and communication in order to improve the management unsafe situations and, as a result, reduce adverse events.

Although several studies have been carried out to evaluate the effectiveness of CRM in health care [22,23], none were conducted at an ICU. Rabøl and colleagues [24] conducted a systematic review about the reported effects of CRM training in health care. They found that the first reaction to the training was very positive and that attitudes changed in favour of the CRM principles. For instance, France and colleagues [25] reported that trainees indicated that CRM has the potential to increase patient safety and quality of care. However, going one step further, looking at behavioural change and the impact of the training on the organisational level (e.g. reduced number of bed days per patient), the results are less straight forward. For example, McCulloch and colleagues [26] found an increase of the use of non-technical skills for nurses, but not for anaesthetists and surgeons.

There are several reasons for these inconsistent findings, which stipulate the necessity for the present research. It can be argued that a lack of consistent findings on a behavioural and organizational level is due to weak study designs. Most of the studies evaluate the effect of CRM within six months [24], which is a relatively short period for an innovation to be completely adopted and to become part of the daily routine [27]. In addition, most evaluations rely on a pre- and post training comparison, but do not include a control group [24]. In some studies the trained and non-trained participants were not separated in data gathering. Furthermore, observations have hardly ever been used to measure behavioural change, despite the high validity of this method as it measures behaviours when they actually occur.

Therefore the aim of the study is to determine the effectiveness and cost-effectiveness of CRM on attitude, behaviour and organization one year after the training. In order to reach this goal, we will use a multi-method approach using questionnaires, direct observations, and interviews. Trained ICUs will be compared with matched control units using pre- and post measurements. The purpose of the present article is to describe the study protocol and the underlying choices of this evaluation study of CRM in the ICU in detail.

## Methods/Design

### Design and setting

Ideally, a CRM training should be evaluated in a large multicenter trial [28]. In our case, time and money were limited, thus we looked for an alternative way to control for institutional variation. We chose for a paired controlled trial, with one pre-test and two post test measurements. Three pairs of comparable ICUs were selected out of a predefined cluster of eligible medium sized units (10 to 16 beds and 55 to 88 employees) of non-academic teaching hospitals. This type of ICU was chosen because these are large enough to form an independent unit, yet small enough to train all of the staff. Moreover, most ICUs in the Netherlands belong to this category or aim to accomplish this. Per pair one ICU received the training directly after the pre-test measurement and one ICU served as a control group. The participating hospitals had on average 641 beds and are located in an urban environment.

Data collection took place from November 2009 until May 2010 (pre-test), June 2010 until September 2010 (post-test 1), and in November 2010 until May 2011 (post-test 2). In each measurement period data were simultaneously collected for the intervention and control unit, and consecutively for all pairs. Per pair each measurement period took 7 to 9 weeks. The intervention units received the training directly after the pre-test. The first post measurement was conducted three months after the training and the second post-measurement followed one year after the training.

ICUs that fitted the profile of a possible intervention unit (i.e. hospital type; ICU size, level and staffing; closed format; taking part in the Dutch National Intensive Care Evaluation (NICE) registry [29]) were approached to participate in the present study (*n *= 12). Most of these ICUs were interested in CRM but unable to fulfil financial needs to start within the study period. Four ICUs were willing to act as an intervention unit and able to pass formal barriers, like finance and organisational arrangements. Of these four, one unit served as a pilot unit, and the other three units served as the intervention group in the main study.

The remaining eight ICUs that fitted the profile were assessed to determine whether they could be matched to one of the intervention ICUs and act as control unit. This assessment comprised a structured conversation with the medical head and team leader and a measurement of the patient safety culture of all the IC-staff by means of the patient safety culture questionnaire [30], which was assessed in the intervention units as well. Important determinants in the matching procedure were the number of beds of the ICU, the number of ICU physicians (fte's), urban or rural area, the perception of patient safety, and the frequency of event reporting. The ICUs that were most similar to one of the intervention ICUs were matched to that ICU and served as a control unit. A total of three pairs of ICUs participated in our study.

The study was approved by the Ethical Committee of the VU University Medical Centre and is in accordance with Dutch privacy regulations. The trial is registered in the Dutch Trial Registration record NTR1976.

### Pilot study

A pilot study was conducted in one ICU with the aim to test whether the planned measurements were organisationally and logistically feasible. Furthermore, the pilot offered a chance for the researchers to get more acquainted with the CRM training as well as with the general daily routine at an ICU. The results of the pilot study indicated that the training was well received and that the planning of the measurements was realistic. Some measurements needed a bit more refinement. For instance, some important verbal behaviours were added to the observation form (e.g. the participant asks for input). Furthermore, the pilot indicated that a CRM change team was important to follow up the plans of action resulting from the training. It was suggested to include three or four IC employees with different backgrounds (e.g. a nurse, an ICU physician, a manager) in the change team. Therefore, in the main study we stimulated to form this change team during the training.

### Intervention: Crew Resource Management training

A commercial vendor of CRM, QST Safe Skies, was contracted to deliver the training. This vendor has much experience with CRM trainings in the aviation sector, as well as in health care. Before the training, all ICU personnel was informed about the study and the training by means of an oral presentation and an information leaflet. Contact between the vendor and the researcher was kept to a minimum during the period in which the ICUs were trained.

The training was classroom based and consisted of a class education session of two consecutive days from 9 till 17 o'clock. Due to a maximum of 15 participants per session, several trainings were organized to educate all members of the IC staff. To limit the period between the training of the first and the last group, every week at least one group was trained. It was made sure that in each group all professions were represented.

The main objectives of the training sessions were to create awareness regarding the threats of suboptimal performance and ways to recognize these threats and prevent their negative consequences. To establish this goal, the participants were educated about CRM concepts and principles, discussed their own experiences with each other, and developed ready to use ideas, all in a setting of trust and openness.

**Figure 1:**
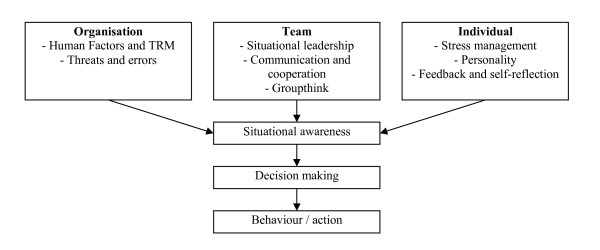
**Schematic representation of the structure of the CRM training**.

With situational awareness as a starting point to identify pitfalls and opportunities for enhancement of the quality of care, several topics were discussed on an organizational, team, and individual level (see Figure 1 and Table 1). Each topic was first introduced by describing the global working mechanisms. This was followed by the risks associated with the specific topic and a suggested approach to overcome these risks. Exercises were used to illustrate or highlight some of the key points. For instance, communication was first theoretically discussed using the sender-receiver model [31]. This was followed by a discussion about what can go wrong in this communication process. To further illustrate communication flaws participants heard a story which they then had to repeat to another person. This showed how quick people forget or even alter parts of a message. Near the end of this part of the training solutions were given to overcome the risks and pitfalls regarding communication (e.g. verify with your sender whether you understood the message correctly).

**Table 1:** A specification of the most important models and theories that were discussed during the CRM training

SHELL-model [62,63]	The SHELL-model emphasizes that human error develops during the interaction between a person (central Lifeware) and the other components of the model, which are Software, Hardware, Environment and other Lifeware.
Swiss Cheese Model [37]	The Swiss Cheese Model of Reason distinguishes different layers, or 'slices', that act as defences or barriers to local hazards. Each layer has flaws, or 'holes', due to active failures and latent conditions. An accident opportunity occurs when different holes line up.
Human Factor Analysis Classification Model [64]	The Human Factor Analysis Classification Model is a framework based on the Swiss Cheese model of Reason and identifies and classifies human causes of error. It defines the 'holes in the cheese'.
Sender-Receiver model [31]	The sender sends a (non-)verbal message to the receiver. How this message will be received depends on the content (the objective aspect), the oblivious added information (the expressive aspect), the relation between the sender and receiver (the relational aspect) and the influence that the sender wants to have on the receiver (the appealing aspect).
Situational Leadership [65]	This model is used to illustrate different styles of leadership and their effectiveness (e.g. 'push' and 'pull' strategy or 'relation' versus 'tasks' oriented leadership). Different situations demand different styles to get the desired outcome
Situation awareness [66]	Situational awareness is 1) the perception and the comprehension of the meaning of environmental elements within a volume of time and space, and 2) the projection of their status and 3) the possible consequences in the near future.
The Johari window [67]	The Johari window is an assessment of the self by yourself and others. There are things that you know and don't know about yourself and there are things that others know and don't know about you. To optimise performance it is necessary to enhance the knowledge of yourself and diminish the part that you and others don't know.
Groupthink [68]	Groupthink is a state of mind of a cohesive group with deeply involved members in which an unanimous decision is more important than to appraise alternatives. There are eight classic symptoms of groupthink: 1) Illusions of invulnerability; 2) Rationalizing warnings; 3) Unquestioned belief in morality of the group; 4) Stereotyping those who are opposed to the group; 5) Direct pressure to conform; 6) Self censorship; 7) Illusions of unanimity amongst group members; 8) Mind guards shielding for dissenting information.
Stress (management)	According to the classic Yerkes Dodson law the relation between stress and performance can be described as an inverted U-shaped pattern. It is vital for performance to maintain the most optimal stress level, therefore to much or to little stress has to be prevented (e.g. through anticipation, mental and physical preparation, mutual trust) or be managed by focusing on the problem or changing emotion, thoughts or behaviour that enhances stress.
Dangerous attitudes	To illustrate how personality can influence decision making five prototypes of dangerous attitudes are discussed. These prototypes are: the anti-authority (will not comply to any rule); the impulsive (acts directly without thinking it through); the invulnerable (thinks that accidents happen only to other people); the macho (wants to prove him/herself in any circumstance); the drop-out (thinks that he/she does not matter).

There were two ways in which CRM concepts were translated into ready to use ideas. At the beginning of the training a discussion about the team roles of different professions in an ICU was carried out. These roles were interactively defined and written down on a sheet. These sheets were readily accessible for modification or adding content when something new was learned. At the end of both the first and second day, plans of action on organizational, team and individual level were formulated by the participants.

A CRM change team was formed in each trained ICU to stimulate and facilitate the implementation of CRM initiatives after the training had ended. This team consisted of enthused representatives of all the professions and management which. The plans of actions of all the training sessions formed the starting point. Who and how many people exactly joined the change team was different for each ICU. Part of the training is that the two CRM instructors offer their help as consultant for one day or two day parts after the ICU was trained. It was up to the change team how to utilize this help (e.g. get organised; implement changes; reiterate theory).

### Framework of analysis and data collection

The present study uses the evaluation framework for training programmes of Kirkpatrick [32] to determine the effectiveness and cost-effectiveness of CRM training. This framework comprises four levels of evaluation and is often applied in the CRM literature [17,24]. The first level is the reaction of the participant to CRM training. This is followed by the level of learning, which includes the gaining of new knowledge or skills and the constitution of new attitudes. The third level entails whether CRM changes behaviour. The fourth level is the organizational impact, for instance a decrease in the number of adverse events as a result of CRM. Each level is assessed with different measurements at the different data collection periods (see Table 2). In addition to the levels of Kirkpatrick, barriers and facilitators for successful implementation of CRM are assessed, to gain insight in the change process.

**Table 2:** Overview of the measurements for each level of Kirkpatrick

		Measurement properties				Data collection period			
Measurements per Level of Kirkpatrick		# Dimensions	# Items (*k*)	Scale	Fill out time (min)	Baseline	After the training	1st Follow-Up	2nd Follow-up
**I**	**Reaction to the training programme**								
	- End-of-course critique questionnaire*	n/a	21	5-Likert	5		X		
	- Evaluation questionnaire*	n/a	13	5-Likert	5			X	
**II**	**Learning from the training programme**								
	- SafeTeamA questionnaire (attitude towards non-technical skills)	7	43	5-Likert	10	X			X
**III**	**Behavioural change as result of the training**								
	- SafeTeamB questionnaire (perception of the use of non-technical skills)	7	39	5-Likert	10	X			X
	- Process control questionnaire	5	28	Yes/No	5			X	X
	- Observation of non-technical skills	6	30	Count	n/a	X			X
	- Interview implementation progress*	n/a	n/a	n/a	n/a		X	X	X
**IV**	**The impact of the training on the organisation**								
	- - COMPaZ (Patent safety culture questionnaire)	11							
	- Error culture questionnaire	11	47	5-Likert	10	X			X
	- Job satisfaction and affective commitment to the ICU	7	18	5-Likert	5	X			X
	- Intake questionnaire	n/a	n/a	n/a	n/a	X			X
	- Routine administration data	n/a	n/a	n/a	n/a	Continuous			

* These measurements were only collected in the intervention groups

n/a = not applicable

All measurements were administered simultaneously in each pair of intervention and control unit, except for the questionnaires regarding reaction to, and evaluation of the training. All measurements are described below in detail.

### Measurements - Questionnaires

#### End-of-course critique

The End-of-Course Critique (ECC) of Grogan and colleagues [22] was used to assess the reaction immediately after the training and assessed the perceived relevance and utility of the specific topics covered in the CRM training (e.g. 'The lecture about 'Human Factors' was relevant and useful'). The ECC consists of 21 statements that have to be rated on a 5-point scale varying from 'strongly disagree' to 'strongly agree' and one open end question.

#### Evaluation questionnaire

This questionnaire was used to assess the extent to which the training altered the awareness regarding CRM topics in their daily work, like the influence of personal and environmental factors on performance. Furthermore, it assesses to what extent the participant felt that there was more situational awareness and enhanced patient safety in the ICU as a result of the training. This was measured with thirteen statements that had to be rated on a 5-point scale, varying from 'not at all' to 'fully applicable'.

In addition to these items, the participant was presented with possible reasons why they did or did not use CRM after the training. A set of 24 reasons were derived from implementation literature [27] and the pilot study. The participant could tick the reason(s) which applied to them (e.g. 'I have no time for CRM' or 'I am convinced that CRM is relevant').

#### SafeTeam questionnaire

The SafeTeam questionnaire is a newly developed questionnaire which is partly based on the Operating Room Management Attitudes Questionnaire (ORMAQ) [33], specifically its items on teamwork and information sharing. Other items were newly developed, derived from insights on speaking up [34] and error management [35]. The SafeTeam contains two sections with seven dimensions each (see Table 3). The A-section assesses attitudes regarding behaviours emphasized in the CRM training. The B-section measures self-reported behaviour regarding CRM principles. This distinction between attitudes and actual behaviour is unique in the setting of CRM evaluations. Both questionnaires use a 5-point Likert scale as answering scale, varying from 'not at all' to 'fully applicable'. The psychometric properties of the SafeTeam will be assessed during this study.

**Table 3:** Dimensions and sample items of the two sections of the SafeTeam questionnaire

Dimension	Sample items	
	SafeTeamA	SafeTeamB
	***In my opinion***...	***In this ICU***...
Information sharing	team members should ask questions if something is unclear	I ask questions when something is unclear to me
Feedback seeking & giving	every team member should be able to give feedback	everyone is minding its own business*
Teamwork	it is a bad idea when members of a team interfere with each others task*	we work as a true team
Situational awareness	we should as a team constantly check if something unusual occurs during the treatment of a patient	we discuss as a team the possibility that something unusual occurs
Speaking up	someone should speak up when he or she notices that a team member is not alert	I speak up when I notice that one of the team members is not alert
Perceived infallibility	it is easy to make mistakes when the tension is high*	my performance is less when I am stressed or tired
Leader coaching	a successful treatment depends on the competence of the physician	there is a significant difference in status between team members

*Reversed coded

#### Process control questionnaire

The process control questionnaire is an abridged version of the Tripod survey [36] and queries about daily work circumstances that may result in substandard acts, or active failures [37]. These circumstances are called basic risk factors. Groeneweg distinguishes 11 of these basic risk factors, six of which are specific for the branch in which the Tripod is developed (i.e. the oil industry) and five are generic. These five generic risk factors regard training, communication, organisation, procedures, and incompatible goals. If these factors are not managed properly, they can start a process that can result in a substandard situation. For instance, when existing guidelines or instructions are not available or of insufficient quality, the chance on non-adherence to these guidelines increases, making procedures a risk factor.

In the present study relevant items in the context of CRM evaluation were selected for each of the generic basic risk factors, thereby reducing the number of items from 75 to 28. For each of the statements participants were asked whether they represented their experiences of the last six months (e.g. 'I could not find the information that I needed to accomplish my task'). The participant could answer 'yes', 'no' or 'do not know'.

#### Patient safety culture

The COMPaZ questionnaire [30] was used to measure the patient safety culture in the ICU. The COMPaZ is the translated Dutch version of the Hospital Survey on Patient Safety Culture (HSOPS) [38]. During translation and validation the COMPaZ was slightly altered from the HSOPS which resulted in 11 dimensions instead of 12 by combining two dimensions of the HSOPS and removing two items. Both questionnaires have successfully been used in previous research [30,38-40].

The COMPaZ consists of 40 items that assess the 11 dimensions. Each item posits a statement that has to be rated on a 5-point scale varying from 'strongly disagree' to 'strongly agree' or 'never' to 'always'. In addition, the COMPaZ comprises a subjective rating of the quality of patient safety in the ICU and incident reporting over the last year.

#### Error culture

The Error Culture Questionnaire (ECQ), developed and validated by Van Dyck [35,41] was used to assess shared attitudes towards, and common responses to error on unit level. According to Van Dyck error culture can be split up into four dimensions, i.e. mastery (trying to overcome errors by learning, analysing and correction), aversion (a rigid and negative attitude towards error occurrence and their deliberate covering up), social (sharing and helping) and awareness (a general readiness to handle errors). Each dimension consists of two or three scales, with a total of 11 scales for all dimensions. These scales are measured with 47 items. Each item is a statement regarding one of the 11 scales. Participants have to rate to which extend this statement applies to the unit on a 5-point scale, varying from 'not at all' to 'completely'. The ECQ has been successfully used in previous research [35,41,42].

#### Job satisfaction and affective commitment to the ICU

Job satisfaction was measured with the Dutch translation [43] of the job satisfaction dimension of the Occupational Stress Inventory (OCI) [44]. The OCI job satisfaction contains six scales that can be used separately. Three scales were selected for the present study (i.e. satisfaction with (1) the job, (2) the organizational design and structure, and (3) the organizational processes). The other three scales were considered to be of less relevance to a CRM evaluation (i.e. appreciation, personal relations, and rewards). The three scales that were used comprised a total of 12 items, which are to be rated on a 5-point scale, varying from 'strongly disagree' to 'strongly agree'. One additional item was added to ask the participant point-blank how satisfied they are with their job (i.e. "Overall, how satisfied are you with your job?").

Affective commitment was assessed with the Dutch translation [45] of the affective subscale of the three component conceptualization of organizational commitment [46]. The questions were slightly altered to the ICU setting by renaming the term 'organization' to 'ICU' in all of the items. The affective commitment scale comprises 6 statements, which are to be answered on a 5-point Likert scale, varying from 'not at all' to 'fully applicable'.

#### Demographics

Several demographic characteristics were administered, including age, gender, position, tenure, experience with working in the hospital and in the ICU, working hours per week, and whether or not the participant has interaction with patients.

### Observation of non-technical skills

Direct observations were used to determine the use of non-technical skills by the IC staff who had direct contact with patients. To assess non-technical skills, an observational model of the Royal Dutch Airlines was used (i.e. SHAPE) [47], which was adjusted for health care (Explicit Professional Oral Communication measurement (EPOC); development and psychometric results will be published separately).

The EPOC classifies explicit professional oral communication of an observed person into six dimensions; assertiveness, working with others, task-oriented leadership, people-oriented leadership, situational awareness, and planning and anticipation. Each dimension is subdivided into several concrete verbal behaviours that together represent the dimension. Throughout an observation of 30 minutes an independent observer tallies how often each verbalization is displayed by the observed person, e.g. 'asks for input', 'coordinates tasks', or 'expresses concerns'. Only professional interaction with co-workers of the ICU was tallied, so social talk or conversations with the patients or family were not tallied.

All observers had a non-medical background in social sciences and were trained for four days. During this training the observers learned the definitions of the verbal behaviours and practised observing at an ICU. To ensure that all observers rated behaviour in the same way and were consistent over time during the data collection period, regular meetings were organized to discuss complex cases. Additionally, 8% of observations were double coded by two independent observers contemporaneously. Furthermore, a 17 minute video of an ICU nurse was used to check the observers' consistency over time. Blinding observers for intervention status of a unit was not possible. Therefore, medical staff was instructed not to discuss the training or CRM issues with the observers and the observers were kept ignorant of the content of the training.

The ICU staff was observed during daily practice, preferably two or three times on different days. All observations took place between 7 am and 7 pm. Each observation had a duration of 30 minutes. At the end of each observation the observed person was asked to fill out the NASA Task Load Index (NASA-TLX) [48] to measure the perceived workload during the observation. The observer independently scored workload as well. Next, the observer filled out which tasks the observed person had done during the thirty minutes of observation, whether there were enough possibilities for professional communication to display non-technical skills, what the level of care the patient received, the number of professional interactions, and with whom.

### Other Measurements

#### Interview implementation progress

Semi-structured interviews were conducted directly after the training and again one year later, to assess if and how CRM had been implemented and what, if any, the stimulating and hindering factors were in this process. Directly after the training the first interview was conducted with one or two persons who introduced CRM to the ICU. The aim of this interview was to discover how CRM came and stayed on the agenda, whether a change team was formed, whether there had been contact with the CRM trainers prior to the training, and whether and how CRM was embedded with existing processes or structures at the unit.

One year after the training, during the second follow-up data collection period, an interview was held with the chairman of the change team. The aim of this interview was to examine whether, and if so, in what ways the ICU had actually implemented CRM after the training. Furthermore, it was asked what their present CRM initiatives were, and what the future directions were. As in the first interview there was special attention to factors that enhanced or hindered successful implementation of CRM. In addition, this second interview aimed to examine the tangible effects of CRM as well as to document the 'do's' and 'don'ts' for future CRM trained departments.

#### Adverse events

Adverse events were assessed using the 'top 9' of adverse outcomes as defined by the adverse outcome committee of the Dutch Association of Intensive Care [49], which are (1) Myocardial infarction; (2) Cardiac arrest, (3) Pneumothorax; (4) Cerebrovascular accident; (5) Critical illness neuro-myopathy; (6) Airway related problems except tracheotomy related problems; (7) Tracheotomy related complications; (8) Problems with vascular access; (9) Bleeding in the proximal and distant digestive tracts. These adverse outcomes were measured using an electronic registration form which was integrated with the digital medical record. Registration was done by the IC physicians as part of the medical record.

#### Patient outcomes

Patient characteristics were registered following current registration standards from the Dutch National Intensive Care Evaluation study [29]. These data were obtained from routine administrative systems following strict definitions and quality checks [50]. Baseline characteristics were collected, using these systems, as defined in the minimal dataset of NICE, which includes general characteristics, such as age, sex, acute and chronic diagnoses, number of admissions, mortality and standardized mortality ratio, Variable Life-Adjusted Display curve, start time and end time of mechanical ventilation, and discharge data from the ICU and hospital. Furthermore, several scoring systems were used to assess the severity of disease(s) and life expectancy of the patient, such as APACHE II [51], APACHE IV [52] and SAPS II [53]. These scores were used to adjust for differences in patient mix. All patient data for this study are anonymous.

#### Additional ICU data - Intake questionnaire

The intake questionnaire assessed basic information about the ICU and was filled out by the head of the ICU. It assessed the number of ICUs within the hospital and the corresponding number of beds per ICU, the total of full time employees, the number of permanent (more than six months) and temporary (less than six months) staff, the percentage of sick leave, participation in NICE registration, the teaching possibilities, the method and use of incident reporting, and the level of the ICU. ICU level refers to the complexity of care that the ICU can manage, varying from a close watch of critical patients for a short number of days (level one) to complex treatment that requires advanced technology and 24 hours per day availability of IC physician (level three). Finally, the intake comprised a question whether the ICU has a closed (specialized IC physician as main clinician) or an open format (the referring physician as main clinician).

### Statistical analyses

The collected data will be checked for completeness and the characteristics and frequency of missing data will be described. Descriptive statistics will be used to describe baseline characteristics of patients, staff and ICU. Comparability of paired intervention and control ICUs will be assessed by comparing baseline data and structural indicators (e.g. number of beds). The effect of the CRM training will be assessed by comparing the before and after measurements of the intervention and control ICUs on all levels of Kirkpatrick's evaluation framework. Changes in patient safety culture, attitude and teamwork behaviour will be described and tested with the ANOVA procedure for repeated measures. This will be done on an individual level with adjustment for the unit. With the observations it is plausible that the different observations within one person will cluster with each other. To control for this clustering, a multilevel analysis will be applied with additional adjustment for the unit. Changes in patient outcomes or incidence of adverse outcomes during the follow-up will be assessed using linear regression analysis while adjusting for case-mix differences and clustering within an ICU.

### Cost-effectiveness analysis

An economic evaluation will be carried out from a societal perspective and according to the Dutch guidelines for costing in economic evaluations [54,55]. The costs of CRM training will be assessed bottom-up, based on personnel time, material, housing and travel costs spend on the training. Direct medical costs of hospital stay will be assessed by multiplying the number of bed days in- and outside ICU with standard cost-prices from a societal perspective. If relevant, costs of extra interventions related to adverse outcomes during ICU stay will be included.

To assess the cost-effectiveness of CRM training compared to no training with regard to patient safety at ICUs, the incremental costs per prevented adverse outcome will be computed. In addition a cost-benefit analysis will be performed to compare incremental costs of training with incremental costs of hospital stay.

## Interpretation of the results

**Figure 2:**
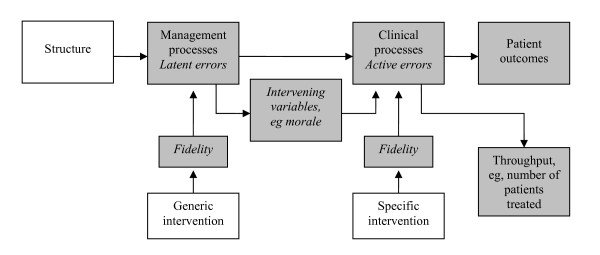
**Causal chain linking interventions to outcomes **[56].

To describe the expected effects of the CRM training the causal chain of Brown [56] can be used (see Figure 2). This model is based on Donabedian's [57] distinction between structure, process and outcome. Structure, the exogenous factors that cannot be completely determined by managers within the organization, influences the endogenous processes within the organization. These processes in turn affect the outcomes and throughput of the organization. According to Brown [56] interventions can influence the process component of this model. He distinguishes two types of interventions: A generic and a specific intervention. A generic intervention is directed at the management or organizational processes of an organisation. A specific intervention focuses on clinical processes. This distinction can be compared to the latent and active failures of Reason [37]. Intervening variables, like morale and culture, connect the management and clinical processes.

Prerequisite for a successful intervention is that the fidelity is high. The fidelity of an intervention is the extent to which it is executed as it was supposed to be executed [58] or as Brown [59] states: "Did it do what was said on the can?" (p.172). We expect that the training has a high fidelity. First of all because the intervention ICUs are highly motivated to receive the training as they invested money and staff time. Furthermore, the training is well developed through previous experience of the instructors. This is also illustrated by the first reaction of the participants of the pilot study, of which over 87% (*n *= 71) stated that the presentations and exercises were relevant and useful.

When CRM is positioned in the causal chain, it can be labelled as a generic intervention that generates specific interventions. By raising awareness of, and creating a shared perspective on, the threats and opportunities in their daily work processes, it enables personnel to recognise strengths and weaknesses [60]. It is expected that this will result in specific interventions to improve these weaknesses and maintain the strengths. For instance, the trained ICU staff can apply the CRM lessons about communication to develop a checklist for clinical handovers in order to minimize miscommunication in this particular situation.

It is expected that the training will result in changes in the intermediate variables of the causal chain, which will result in specific improvement actions in practice. First, small interventions may be implemented to gain rapid success, i.e. installing and using a white board for communication. It may take more time to implement more complex and structural changes, such as implementing a protocol for safe patient transport. The ultimate goal of the training is that CRM principles are structurally embedded in the organization of the ICU and adopted as an integral part of the patient safety and error management culture.

## Concluding remarks

The present study design is developed to assess the effects of CRM in the ICU, as well as to describe the process that explain such effects. What makes this study unique relative to other CRM evaluations is the combination of the long follow up of one year, the assessment of behavioural change with observations, and the use of matched control units. As recommended and used by several authors [17,23] the framework of Kirkpatrick is employed to distinguish different levels of effect. Besides the observations, a mix of different instruments is used in order to explain the effect on the levels of Kirkpatrick's framework. The matched control units protect the study against secular trends and sudden changes [61].

This study design pays particular attention to practicalities of implementing CRM, by incorporating the assessment of barriers and facilitators to follow up on CRM initiatives developed during the training. This will increase the understanding of the effect of CRM training at the behavioural and organizational level [24]. Furthermore, knowledge on barriers and facilitators will provide a pragmatic start for units that consider training their unit.

Improving the use of non-technical skills of health care professionals in the ICU provides an opportunity to enhance the quality of care and decrease the number of adverse events. CRM appears to take full advantage of this opportunity. It stimulates the individual, as well as the team, to be aware of threats and risks and to manage unsafe situations effectively, for instance by communicating more explicitly. The plans of action resulting from the CRM training provide concrete starting points to implement CRM initiatives, which in turn can create a snowball effect of generic and specific interventions aimed at the improvement of quality and safety of the ICU. These initiatives may improve the management and clinical processes of the unit as well as patient outcomes. By learning from previous research, incorporating new perspectives and keeping an eye on the practical implications, this study design will determine how and to what extent CRM training accomplishes these effects.

## Abbreviations

APACHE: Acute Physiology and Chronic Health Evaluation; CRM: Crew Resource Management; ICU: Intensive Care Unit; NICE: Dutch National Intensive Care Evaluation; SAPS: Simplified Acute Physiology Score

## Competing interests

The authors declare that they have no competing interests.

## Authors' contributions

PK drafted the final manuscript. MdB conceived the design of the study, drafted the initial research proposal and helped to draft the final manuscript. CvD and CW participated in the design of the study and helped to draft the manuscript. All authors read and approved the final manuscript.

## Pre-publication history

The pre-publication history for this paper can be accessed here:

http://www.biomedcentral.com/1472-6963/11/304/prepub
